# Supplementary material for: Network analysis of synovial RNA sequencing identifies gene-gene interactions predictive of response in rheumatoid arthritis
Source: Arthritis Res Ther. 2022 Jul 11;24:166. doi: 10.1186/s13075-022-02803-z (PMC9275048; doi:10.1186/s13075-022-02803-z)
Supplement: Supplementary file 1 — Additional file 1. File including supplementary methods figures, and tables S6-16. [file 13075_2022_2803_MOESM1_ESM.pdf]

## SUPPLEMENTARY MATERIAL

### Supplementary Methods

*Patient cohort*

In total, RNA-Seq data from 94 patients with early, treatment-naïve RA fulfilling the 2010 ACR/EULAR Criteria who had been recruited into the Pathobiology of Early Arthritis Cohort (PEAC) (Lewis et al. 2019; Humby et al. 2019) was used for the current study. 11 samples were removed due to poor RNA quality (n=7) or ungraded histopathology (n=4), thus 83 samples were included in the present study (see Table 1). As previously described (Lewis et al. 2019; Humby et al. 2019), patients were stratified into three distinct synovial histopathological groups: (1) *lympho-myeloid* characterized by lymphoid cell infiltration such as T-cells, B-cells and plasma cells together with myeloid cells; (2) *diffuse-myeloid* with a high prevalence of cells from the myeloid lineage with low numbers in B-cells and plasma-cells; (3) *pauci-immune* identified by lack of infiltrating immune cells associated with stromal cell expansion. After a baseline biopsy (treatment-naïve), patients underwent six months of csDMARD therapy and responsiveness was assessed according to DAS28 EULAR criteria (see Table 2). Clinical outcome measures were determined by clinicians entirely blinded to any synovial biopsy histology and RNA-Seq data. All RNA extraction and RNA-Seq was performed independently of knowledge of clinical outcome.

*Network analysis*

The study pipeline is summarised in Figure 1A. Samples from the PEAC cohort were initially categorised into histopathological and treatment response groups with separate analyses run for each type of classification. When using the DAS28 EULAR response categorisation samples classified as *moderate responders* were excluded to allow cleaner identification of response signatures. After Variance Stabilizing Transformation (VST) of the count data via DESeq2, the mean normalised gene

expression profile was derived for each group. In 2016 Alaimo et *al.* merged information from four repositories to generate a network of 10,537 interactions obtained from KEGG (Kanehisa and Goto 2000), mirTARbase (Hsu et al. 2011), miRecords (Xiao et al. 2009) and transmiR (Tong et al. 2019). In these curated repositories gene-gene interactions include protein-protein interactions which have been reported from experiments in the literature including co-immunoprecipitation, yeast-2-hybrid and direct molecular biology studies, as well as gene-gene interactions based on integration of microRNA and transcriptome data. Such an extensive network was exported via the *exportgraph* function in MITHrIL (Alaimo et al. 2016) and replicated for each group. To generate networks specific for each histopathological or response subgroup, weights were assigned to gene nodes, equal to the computed mean gene expression across that particular subgroup. Nodes representing genes whose mean expression was below the 75th percentile of the entire VST matrix were removed from each network. This cut-off level was determined following a percolation analysis. Commonly used in statistical physics and mathematics, percolation describes the behaviour of network properties when a certain percentage of nodes or links are removed(Sahini and Sahimi 1994). In this implementation, the percolation threshold was optimised to maximise the number of statistically significant differential interactions.

In a second filtering step remaining interactions in common between two or more group networks were removed, to obtain group-specific interactions for each network.

The resulting set of networks presented easily identifiable clusters that were enriched by means of ClueGO (Bindea et al. 2009) (v.2.5.5). Four repositories were selected for enrichment: KEGG (Kanehisa and Goto 2000), REACTOME (Jassal et al. 2020) and two Gene Ontology (GO) databases (Camon et al. 2004), *BiologicalProcess-EBI-UniProt-GOA* and *ImmuneSystemProcess-EBI-UniProt-GOA* at 5 or 6 GO tree levels.

For each cluster, the significant GO term/pathway ($p < 0.05$) containing the highest percentage of cluster genes was annotated in Table 2. Enrichment percentage scores refer to the percentage of genes within a specific cluster which are enriched within a given pathway.

The code used to implement the described network analysis is publicly available on github (https://github.com/elisabettasciacca/DEGGs) and is being prepared for submission as an R package to the Bioconductor repository.

*Robust linear regression interaction analysis*

We then evaluated the statistical significance of single links by estimating the expression of each node interaction of a group network. A robust linear regression model with interaction term was fitted using the rlm function from the MASS (v.7.3) R package:

$$GeneA_i = \beta_0 + \beta_1\ GeneB_i + \beta_2\ Response_i + \beta_3\ GeneB_i * Response_i + \varepsilon_i$$

where $i = 1, \ldots, n$, is the number of samples and $\varepsilon_i$ are random variables.

*Response* is replaced by *Pathotype* in the case of pathotype comparison.

Interaction analysis was performed across all gene nodes present within each network (Table S1).

The ratio of gene pairs whose interaction term *GeneB * Response* was significant was used to predict response (as categorical variable) to csDMARD treatment:

$$Response_i = \beta_0 + \beta_1\ GeneA_i + \beta_2\ GeneB_i + \beta_3\ GeneA_i / GeneB_i + \varepsilon_i$$

Evaluation of possible confounding factors (age, gender, CCP status, pathotype) was investigated by adding putative covariates to the derived predictive models (Table S6-9). None of the additive confounding factors reached statistical significance for their estimates within the linear models, with the exception of *age* in the predictive model for *STAT2-SOCS2*, where this covariate was incorporated.

Models were tested to determine whether the presence of the interaction term expressed by the ratio of the two genes enhance the prediction ability of the model. Fitted models were tested for predictive ability by likelihood ratio test P-value as well as receiver operating characteristic (ROC) curve analysis using the pROC package in R in comparison to the equivalent model formula without the additional gene/gene ratio term.

*Genotyping*

Genomic DNA was isolated from 128 rheumatoid arthritis patients from the wider PEAC cohort. Genotyping was carried out using an Illumina Human CoreExome-24 version 1-0 array, following the manufacturer's protocol. Quality control (QC) on the imputed SNP data was performed using standard procedures outlined by Anderson et *al.* (Anderson et al. 2010). Individuals were excluded if the reported sex did not match the sex assessed by genotype, and samples with elevated missingness rate, outlying heterozygosity rate, outlying ethnicity and relatedness were also excluded. SNPs were excluded if they had a post-imputation INFO score <0.8. Genotype hard calls were set to missing if the posterior probability was <0.9. The data was filtered by minor allele frequency (>0.01), Hardy–Weinberg disequilibrium (p>0.000001) and missing genotype rate (<0.05). SNP genotypes were encoded according to the number of copies of the minor allele.

Principal component analysis (PCA) was performed to find covariates on the genotype data which had been pruned for linkage disequilibrium (LD) ≤0.2 using the SNPRelate package (v1.18.1) (Zheng et al. 2012). A minor allele frequency (MAF) cut-off of MAF ≥0.05 was used to remove variants. After QC, genotyping was available on 73 patients with matched RNA-Seq samples.

*HLA imputation and statistical analysis*

HLA alleles within the major histocompatibility complex (MHC) region in chromosome 6 (Okada et al. 2014; Pillai et al. 2014; Jia et al. 2013) were imputed using HLA-TAPAS (Luo et al. 2021) with a reference panel built by 2,504 individuals from the 1000 Genomes project (Auton and Salcedo 2015).

To statistically assess whether differential recurrence could be observed across pathotypes, CCP status and EULAR response groups, linear regression models were build evaluating the occurrence of the *HLA-DRB1* alleles as a function of the variables. For CCP status (categorical variable with two levels) the p-value of the CCP term was reported. For EULAR response and pathotypes (categorical variables with three levels), a second linear model expressing the *HLA-DRB1* occurrence as a function

of the intercept was built. The two models were compared via ANOVA test and the resulting p-value was reported.

## References

Alaimo S, Giugno R, Acunzo M, Veneziano D, Ferro A, Pulvirenti A. 2016. Post-transcriptional knowledge in pathway analysis increases the accuracy of phenotypes classification. *Oncotarget* **7**: 54572–54582.

Anderson CA, Pettersson FH, Clarke GM, Cardon LR, Morris AP, Zondervan KT. 2010. Data quality control in genetic case-control association studies. *Nat Protoc*.

Auton A, Salcedo T. 2015. The 1000 genomes project. In *Assessing Rare Variation in Complex Traits: Design and Analysis of Genetic Studies*.

Bindea G, Mlecnik B, Hackl H, Charoentong P, Tosolini M, Kirilovsky A, Fridman WH, Pagès F, Trajanoski Z, Galon J. 2009. ClueGO: A Cytoscape plug-in to decipher functionally grouped gene ontology and pathway annotation networks. *Bioinformatics* **25**: 1091–1093.

Camon E, Magrane M, Barrell D, Lee V, Dimmer E, Maslen J, Binns D, Harte N, Lopez R, Apweiler R. 2004. The Gene Ontology Annotation (GOA) Database: Sharing knowledge in Uniprot with Gene Oncology. *Nucleic Acids Res*.

Hsu S Da, Lin FM, Wu WY, Liang C, Huang WC, Chan WL, Tsai WT, Chen GZ, Lee CJ, Chiu CM, et al. 2011. MiRTarBase: A database curates experimentally validated microRNA-target interactions. *Nucleic Acids Res*.

Humby F, Lewis M, Ramamoorthi N, Hackney JA, Barnes MR, Bombardieri M, Setiadi AF, Kelly S, Bene F, DiCicco M, et al. 2019. Synovial cellular and molecular signatures stratify clinical response to csDMARD therapy and predict radiographic progression in early rheumatoid arthritis patients. *Ann Rheum Dis* **78**: 761–772.

Jassal B, Matthews L, Viteri G, Gong C, Lorente P, Fabregat A, Sidiropoulos K, Cook J, Gillespie M, Haw R, et al. 2020. The reactome pathway knowledgebase. *Nucleic Acids Res* **48**: D498–D503.

Jia X, Han B, Onengut-Gumuscu S, Chen WM, Concannon PJ, Rich SS, Raychaudhuri S, de

Bakker PIW. 2013. Imputing Amino Acid Polymorphisms in Human Leukocyte Antigens. *PLoS One*.

Kanehisa M, Goto S. 2000. KEGG: Kyoto Encyclopedia of Genes and Genomes. *Nucleic Acids Res* **28**: 27–30.

Lewis MJ, Barnes MR, Blighe K, Goldmann K, Rana S, Hackney JA, Ramamoorthi N, John CR, Watson DS, Kummerfeld SK, et al. 2019. Molecular Portraits of Early Rheumatoid Arthritis Identify Clinical and Treatment Response Phenotypes. *Cell Rep* **28**: 2455-2470.e5.

Luo Y, Kanai M, Choi W, Li X, Sakaue S, Yamamoto K, Ogawa K, Gutierrez-Arcelus M, Gregersen PK, Stuart PE, et al. 2021. A high-resolution HLA reference panel capturing global population diversity enables multi-ancestry fine-mapping in HIV host response. *Nat Genet*.

Okada Y, Kim K, Han B, Pillai NE, Ong RTH, Saw WY, Luo M, Jiang L, Yin J, Bang SY, et al. 2014. Risk for ACPA-positive rheumatoid arthritis is driven by shared HLA amino acid polymorphisms in Asian and European populations. *Hum Mol Genet*.

Pillai NE, Okada Y, Saw WY, Ong RTH, Wang X, Tantoso E, Xu W, Peterson TA, Bielawny T, Ali M, et al. 2014. Predicting HLA alleles from high-resolution SNP data in three Southeast Asian populations. *Hum Mol Genet*.

Sahini M, Sahimi M. 1994. *Applications Of Percolation Theory*.

Tong Z, Cui Q, Wang J, Zhou Y. 2019. TransmiR v2.0: An updated transcription factor-microRNA regulation database. *Nucleic Acids Res* **47**: D253–D258.

Xiao F, Zuo Z, Cai G, Kang S, Gao X, Li T. 2009. miRecords: An integrated resource for microRNA-target interactions. *Nucleic Acids Res* **37**: D105–D110.

Zheng X, Levine D, Shen J, Gogarten SM, Laurie C, Weir BS. 2012. A high-performance computing toolset for relatedness and principal component analysis of SNP data. *Bioinformatics*.

## Supplementary Tables

Supplementary tables S1-5 are provided in xlsx format in *Additional file 2*.

| model | additional covariate (p-value) | STAT2 (p-value) | SOCS2 (p-value) | STAT2:SOCS2 (p-value) |
|---|---|---|---|---|
| Response~STAT2+SOCS2+STAT2:SOCS2 | | 0.02 | 0.021 | 0.026 |
| Response~Age+STAT2+SOCS2+STAT2:SOCS2 | 0.047 | 0.01 | 0.011 | 0.010 |
| Response~Gender+STAT2+SOCS2+STAT2:SOCS2 | 0.494 | 0.014 | 0.014 | 0.017 |
| Response~CCP+STAT2+SOCS2+STAT2:SOCS2 | 0.442 | 0.018 | 0.019 | 0.022 |
| Response~Pathotype+STAT2+SOCS2+STAT2:SOCS2 | 0.917 | 0.014 | 0.014 | 0.017 |

**Table S6.** Table of p-values associated to each term of the robust linear model for *STAT2-SOCS2*. Four confounding factors have been systematically added to assess their potential role in response prediction.

| model | additional covariate (p-value) | PPP2R3B (p-value) | AKT1 (p-value) | PPP2R3B:AKT1 (p-value) |
|---|---|---|---|---|
| Response~PPP2R3B+AKT1+PPP2R3B:AKT1 | | 0.004 | 0.002 | 0.004 |
| Response~Age+PPP2R3B+AKT1+PPP2R3B:AKT1 | 0.186 | 0.079 | 0.048 | 0.081 |
| Response~Gender+PPP2R3B+AKT1+PPP2R3B:AKT1 | 0.356 | 0.246 | 0.17 | 0.246 |
| Response~CCP+PPP2R3B+AKT1+PPP2R3B:AKT1 | 0.369 | 0.185 | 0.13 | 0.186 |
| Response~Pathotype+PPP2R3B+AKT1+PPP2R3B:AKT1 | 0.719 | 0.164 | 0.111 | 0.164 |

**Table S7.** Table of p-values associated to each term of the robust linear model for *PPP2R3B-AKT1*. Four confounding factors have been systematically added to assess their potential role in response prediction.

| model | additional covariate (p-value) | CAMK2D (p-value) | NOS3 (p-value) | CAMK2D:NOS3 (p-value) |
|---|---|---|---|---|
| Response~CAMK2D+NOS3+CAMK2D:NOS3 | | 0.01 | 0.009 | 0.008 |
| Response~Age+CAMK2D+NOS3+CAMK2D:NOS3 | 0.301 | 0.031 | 0.027 | 0.025 |
| Response~Gender+CAMK2D+NOS3+CAMK2D:NOS3 | 0.642 | 0.067 | 0.067 | 0.062 |
| Response~CCP+CAMK2D+NOS3+CAMK2D:NOS3 | 0.479 | 0.078 | 0.073 | 0.069 |
| Response~Pathotype+CAMK2D+NOS3+CAMK2D:NOS3 | 0.205 | 0.019 | 0.019 | 0.016 |

**Table S8.** Table of p-values associated to each term of the robust linear model for *CAMK2D-NOS3*. Four confounding factors have been systematically added to assess their potential role in response prediction.

| model | additional covariate (p-value) | PIK3CD (p-value) | ATP1B1 (p-value) | PIK3CD:ATP1B1 (p-value) |
|---|---|---|---|---|
| Response~PIK3CD+ATP1B1+PIK3CD:ATP1B1 | | 0.052 | 0.034 | 0.047 |
| Response~Age+PIK3CD+ATP1B1+PIK3CD:ATP1B1 | 0.082 | 0.063 | 0.038 | 0.057 |
| Response~Gender+PIK3CD+ATP1B1+PIK3CD:ATP1B1 | 0.676 | 0.084 | 0.068 | 0.083 |
| Response~CCP+PIK3CD+ATP1B1+PIK3CD:ATP1B1 | 0.461 | 0.085 | 0.069 | 0.083 |
| Response~Pathotype+PIK3CD+ATP1B1+PIK3CD:ATP1B1 | 0.946 | 0.081 | 0.064 | 0.078 |

**Table S9.** Table of p-values associated to each term of the robust linear model for *PIK3CD-ATP1B1*. Four confounding factors have been systematically added to assess their potential role in response prediction.

| | **Lymphoid (N=43)** | **Myeloid (N=17)** | **Fibroid (N=13)** | **Total (N=73)** | **P value** |
|---|---|---|---|---|---|
| HLA_DRB1_04 | | | | | 0.41 |
| 0 | 23 (53.5%) | 10 (58.8%) | 4 (30.8%) | 37 (50.7%) | |
| 1 | 15 (34.9%) | 5 (29.4%) | 7 (53.8%) | 27 (37.0%) | |
| 2 | 5 (11.6%) | 2 (11.8%) | 2 (15.4%) | 9 (12.3%) | |
| HLA_DRB1_0401 | | | | | 0.63 |
| 0 | 25 (58.1%) | 11 (64.7%) | 5 (38.5%) | 41 (56.2%) | |
| 1 | 14 (32.6%) | 4 (23.5%) | 7 (53.8%) | 25 (34.2%) | |
| 2 | 4 (9.3%) | 2 (11.8%) | 1 (7.7%) | 7 (9.6%) | |
| HLA_DRB1_01 | | | | | 0.10 |
| 0 | 27 (62.8%) | 13 (76.5%) | 12 (92.3%) | 52 (71.2%) | |
| 1 | 16 (37.2%) | 4 (23.5%) | 1 (7.7%) | 21 (28.8%) | |
| 2 | 0 (0.0%) | 0 (0.0%) | 0 (0.0%) | 0 (0.0%) | |
| HLA_DRB1_0101 | | | | | 0.10 |
| 0 | 27 (62.8%) | 13 (76.5%) | 12 (92.3%) | 52 (71.2%) | |
| 1 | 16 (37.2%) | 4 (23.5%) | 1 (7.7%) | 21 (28.8%) | |
| 2 | 0 (0.0%) | 0 (0.0%) | 0 (0.0%) | 0 (0.0%) | |
| HLA_DRB1_13 | | | | | 0.77 |
| 0 | 30 (69.8%) | 11 (64.7%) | 10 (76.9%) | 51 (69.9%) | |
| 1 | 13 (30.2%) | 6 (35.3%) | 3 (23.1%) | 22 (30.1%) | |
| 2 | 0 (0.0%) | 0 (0.0%) | 0 (0.0%) | 0 (0.0%) | |

**Table S10.** Frequency table showing the distribution of the top five most frequent *HLA-DRB1* alleles across pathotypes.

| | CCP pos (N=54) | CCP neg (N=19) | Total (N=73) | P value |
|---|---|---|---|---|
| HLA_DRB1_04 | | | | 0.30 |
| 0 | 26 (48.1%) | 11 (57.9%) | 37 (50.7%) | |
| 1 | 20 (37.0%) | 7 (36.8%) | 27 (37.0%) | |
| 2 | 8 (14.8%) | 1 (5.3%) | 9 (12.3%) | |
| HLA_DRB1_0401 | | | | 0.65 |
| 0 | 30 (55.6%) | 11 (57.9%) | 41 (56.2%) | |
| 1 | 18 (33.3%) | 7 (36.8%) | 25 (34.2%) | |
| 2 | 6 (11.1%) | 1 (5.3%) | 7 (9.6%) | |
| HLA_DRB1_01 | | | | 0.15 |
| 0 | 36 (66.7%) | 16 (84.2%) | 52 (71.2%) | |
| 1 | 18 (33.3%) | 3 (15.8%) | 21 (28.8%) | |
| 2 | 0 (0.0%) | 0 (0.0%) | 0 (0.0%) | |
| HLA_DRB1_0101 | | | | 0.15 |
| 0 | 36 (66.7%) | 16 (84.2%) | 52 (71.2%) | |
| 1 | 18 (33.3%) | 3 (15.8%) | 21 (28.8%) | |
| 2 | 0 (0.0%) | 0 (0.0%) | 0 (0.0%) | |
| HLA_DRB1_13 | | | | 0.46 |
| 0 | 39 (72.2%) | 12 (63.2%) | 51 (69.9%) | |
| 1 | 15 (27.8%) | 7 (36.8%) | 22 (30.1%) | |
| 2 | 0 (0.0%) | 0 (0.0%) | 0 (0.0%) | |

**Table S11.** Frequency table showing the distribution of the top five most frequent *HLA-DRB1* alleles in CCP positive and negative patients.

| | Good (N=24) | Moderate (N=28) | None (N=9) | Total (N=61) | P value |
|---|---|---|---|---|---|
| HLA_DRB1_04 | | | | | 0.214 |
| 0 | 9 (37.5%) | 19 (67.9%) | 3 (33.3%) | 31 (50.8%) | |
| 1 | 13 (54.2%) | 6 (21.4%) | 5 (55.6%) | 24 (39.3%) | |
| 2 | 2 (8.3%) | 3 (10.7%) | 1 (11.1%) | 6 (9.8%) | |
| HLA_DRB1_0401 | | | | | 0.581 |
| 0 | 12 (50.0%) | 19 (67.9%) | 3 (33.3%) | 34 (55.7%) | |
| 1 | 11 (45.8%) | 6 (21.4%) | 6 (66.7%) | 23 (37.7%) | |
| 2 | 1 (4.2%) | 3 (10.7%) | 0 (0.0%) | 4 (6.6%) | |
| HLA_DRB1_01 | | | | | 0.283 |
| 0 | 17 (70.8%) | 17 (60.7%) | 8 (88.9%) | 42 (68.9%) | |
| 1 | 7 (29.2%) | 11 (39.3%) | 1 (11.1%) | 19 (31.1%) | |
| 2 | 0 (0.0%) | 0 (0.0%) | 0 (0.0%) | 0 (0.0%) | |
| HLA_DRB1_0101 | | | | | 0.283 |
| 0 | 17 (70.8%) | 17 (60.7%) | 8 (88.9%) | 42 (68.9%) | |
| 1 | 7 (29.2%) | 11 (39.3%) | 1 (11.1%) | 19 (31.1%) | |
| 2 | 0 (0.0%) | 0 (0.0%) | 0 (0.0%) | 0 (0.0%) | |
| HLA_DRB1_13 | | | | | 0.856 |
| 0 | 17 (70.8%) | 19 (67.9%) | 7 (77.8%) | 43 (70.5%) | |
| 1 | 7 (29.2%) | 9 (32.1%) | 2 (22.2%) | 18 (29.5%) | |
| 2 | 0 (0.0%) | 0 (0.0%) | 0 (0.0%) | 0 (0.0%) | |

**Table S12.** Frequency table showing the distribution of the top five most frequent *HLA-DRB1* alleles across DAS28 EULAR responder groups.

| model | additional covariate (p-value) | STAT2 (p-value) | SOCS2 (p-value) | STAT2:SOCS2 (p-value) |
|---|---|---|---|---|
| Response~STAT2+SOCS2+STAT2:SOCS2 | | 0.02 | 0.021 | 0.026 |
| Response~HLA_DRB1_04+STAT2+SOCS2+STAT2:SOCS2 | 0.607 | 0.031 | 0.032 | 0.039 |
| Response~HLA_DRB1_0401+STAT2+SOCS2+STAT2:SOCS2 | 0.651 | 0.03 | 0.031 | 0.037 |
| Response~HLA_DRB1_01+STAT2+SOCS2+STAT2:SOCS2 | 0.372 | 0.034 | 0.033 | 0.038 |
| Response~HLA_DRB1_0101+STAT2+SOCS2+STAT2:SOCS2 | 0.372 | 0.034 | 0.033 | 0.038 |
| Response~HLA_DRB1_13+STAT2+SOCS2+STAT2:SOCS2 | 0.527 | 0.027 | 0.028 | 0.032 |

**Table S13.** Table of p-values associated to each term of the robust linear model for *STAT2-SOCS2*. Top five most frequent *HLA-DRB1* alleles have been systematically added to the original model to assess their potential role in response prediction.

| model | additional covariate (p-value) | PPP2R3B (p-value) | AKT1 (p-value) | PPP2R3B:AKT1 (p-value) |
|---|---|---|---|---|
| Response~PPP2R3B+AKT1+PPP2R3B:AKT1 | | 0.004 | 0.002 | 0.004 |
| Response~HLA_DRB1_04+PPP2R3B+AKT1+PPP2R3B:AKT1 | 0.452 | 0.134 | 0.081 | 0.137 |
| Response~HLA_DRB1_0401+PPP2R3B+AKT1+PPP2R3B:AKT1 | 0.89 | 0.146 | 0.094 | 0.147 |
| Response~HLA_DRB1_01+PPP2R3B+AKT1+PPP2R3B:AKT1 | 0.05 | 0.231 | 0.134 | 0.236 |
| Response~HLA_DRB1_0101+PPP2R3B+AKT1+PPP2R3B:AKT1 | 0.05 | 0.231 | 0.134 | 0.236 |
| Response~HLA_DRB1_13+PPP2R3B+AKT1+PPP2R3B:AKT1 | 0.752 | 0.196 | 0.131 | 0.198 |

**Table S14.** Table of p-values associated to each term of the robust linear model for *PPP2R3B-AKT1*. Top five most frequent *HLA-DRB1* alleles have been systematically added to the original model to assess their potential role in response prediction.

| model | additional covariate (p-value) | CAMK2D (p-value) | NOS3 (p-value) | CAMK2D:NOS3 (p-value) |
|---|---|---|---|---|
| Response~CAMK2D+NOS3+CAMK2D:NOS3 | | 0.01 | 0.009 | 0.008 |
| Response~HLA_DRB1_04+CAMK2D+NOS3+CAMK2D:NOS3 | 0.809 | 0.028 | 0.026 | 0.024 |
| Response~HLA_DRB1_0401+CAMK2D+NOS3+CAMK2D:NOS3 | 0.495 | 0.009 | 0.008 | 0.007 |
| Response~HLA_DRB1_01+CAMK2D+NOS3+CAMK2D:NOS3 | 0.694 | 0.021 | 0.018 | 0.017 |
| Response~HLA_DRB1_0101+CAMK2D+NOS3+CAMK2D:NOS3 | 0.694 | 0.021 | 0.018 | 0.017 |
| Response~HLA_DRB1_13+CAMK2D+NOS3+CAMK2D:NOS3 | 0.637 | 0.02 | 0.019 | 0.017 |

**Table S15.** Table of p-values associated to each term of the robust linear model for *CAMK2D-NOS3*. Top five most frequent *HLA-DRB1* alleles have been systematically added to the original model to assess their potential role in response prediction.

| model | additional covariate (p-value) | PIK3CD (p-value) | ATP1B1 (p-value) | PIK3CD:ATP1B1 (p-value) |
|---|---|---|---|---|
| Response~PIK3CD+ATP1B1+PIK3CD:ATP1B1 | | 0.052 | 0.034 | 0.047 |
| Response~HLA_DRB1_04+PIK3CD+ATP1B1+PIK3CD:ATP1B1 | 0.721 | 0.11 | 0.08 | 0.101 |
| Response~HLA_DRB1_0401+PIK3CD+ATP1B1+PIK3CD:ATP1B1 | 0.953 | 0.106 | 0.08 | 0.1 |
| Response~HLA_DRB1_01+PIK3CD+ATP1B1+PIK3CD:ATP1B1 | 0.29 | 0.09 | 0.072 | 0.09 |
| Response~HLA_DRB1_0101+PIK3CD+ATP1B1+PIK3CD:ATP1B1 | 0.29 | 0.09 | 0.072 | 0.09 |
| Response~HLA_DRB1_13+PIK3CD+ATP1B1+PIK3CD:ATP1B1 | 0.979 | 0.108 | 0.081 | 0.102 |

**Table S16.** Table of p-values associated to each term of the robust linear model for *PIK3CD-ATP1B1*. Top five most frequent *HLA-DRB1* alleles have been systematically added to the original model to assess their potential role in response prediction.

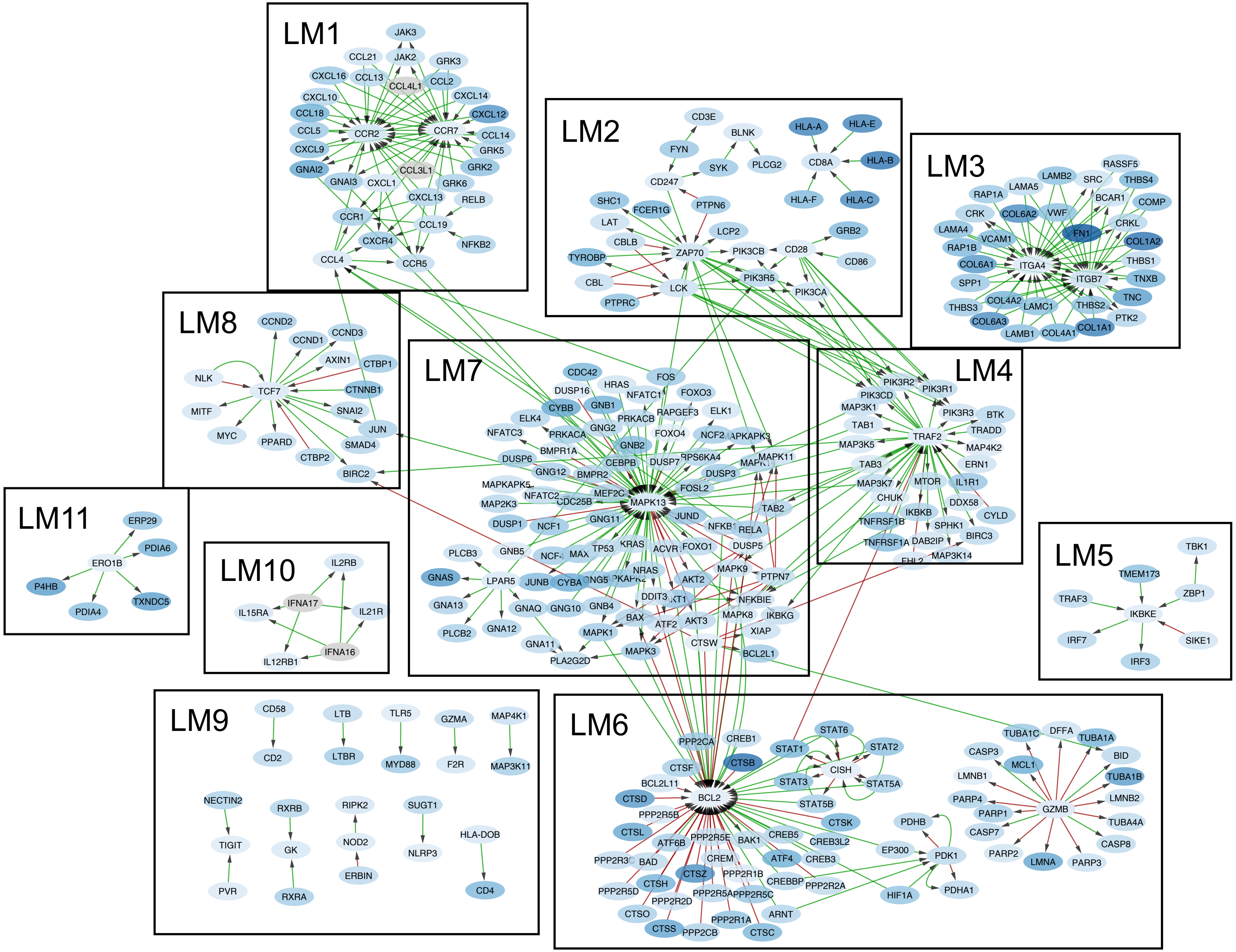


**Figure S1.** Network of specific interactions for the lympho-myeloid pathotype (334 nodes, 469 edges, 11 clusters). The specific links were obtained from 10,537 KEGG interactions filtered by both gene expression and comparison with the diffuse-myeloid and pauci-immune fibroid pathotype. Interactions classified in KEGG as activation links are showed in green. Inhibition links are showed in red.
The most prominent clusters of this network are shown in more detail in Figure 1B.

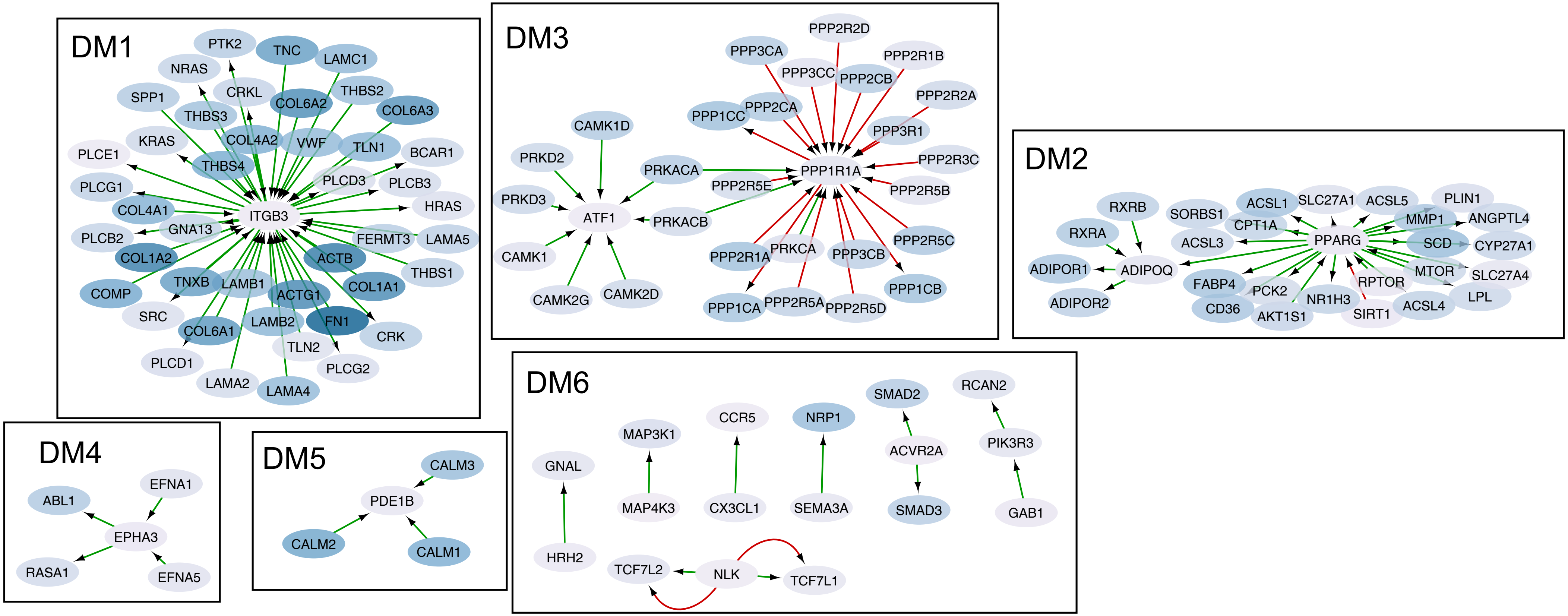


**Figure S2.** Diffuse-myeloid network containing 129 nodes and 98 edges obtained from 10,537 KEGG interactions filtered by gene expression.
Highly expressed links in common with the lympho-myeloid and pauci-immune fibroid pathotype were also removed.
Interactions classified in KEGG as activation links are showed in green. Inhibition links are showed in red.
Six clusters have been identified of which the most prominent are shown in more detail in Figure 2A.

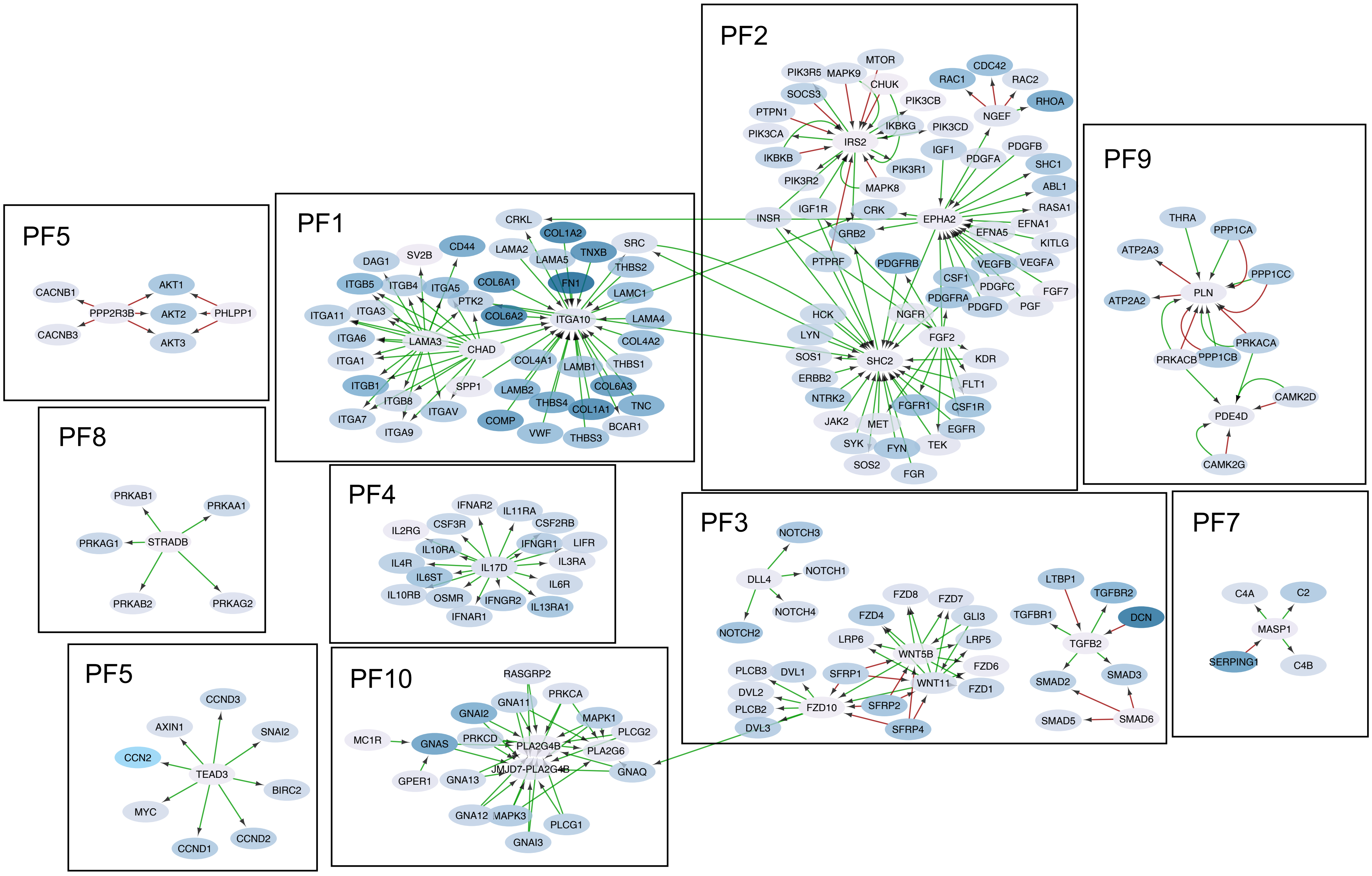


**Figure S3.** Pauci-immune fibroid network showing 229 nodes, 299 links and 10 clusters. Highly expressed links in common with the lympho-myeloid and the diffuse-myeloid groups were removed.
Interactions classified in KEGG as activation links are showed in green. Inhibition links are showed in red.
The most prominent clusters of this network are reported in more detail in Figure 2B.

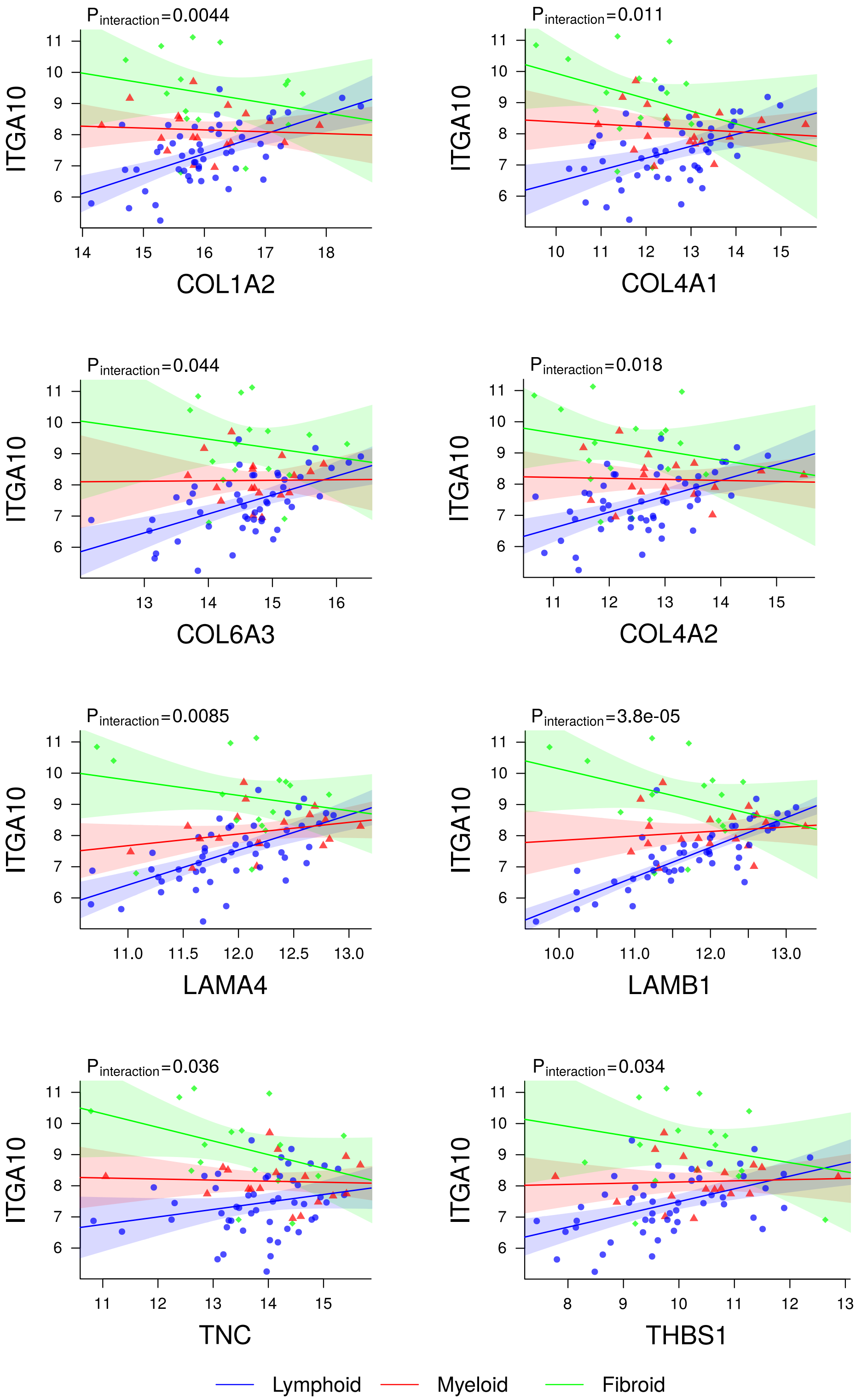


**Figure S4.** Robust linear regression showing interactions of integrin alpha 10 (*ITGA10*) with ECM genes differentiating across pathotypes with the same recurring pattern of correlations. P value of the gene:pathotype interacting term is shown on top.

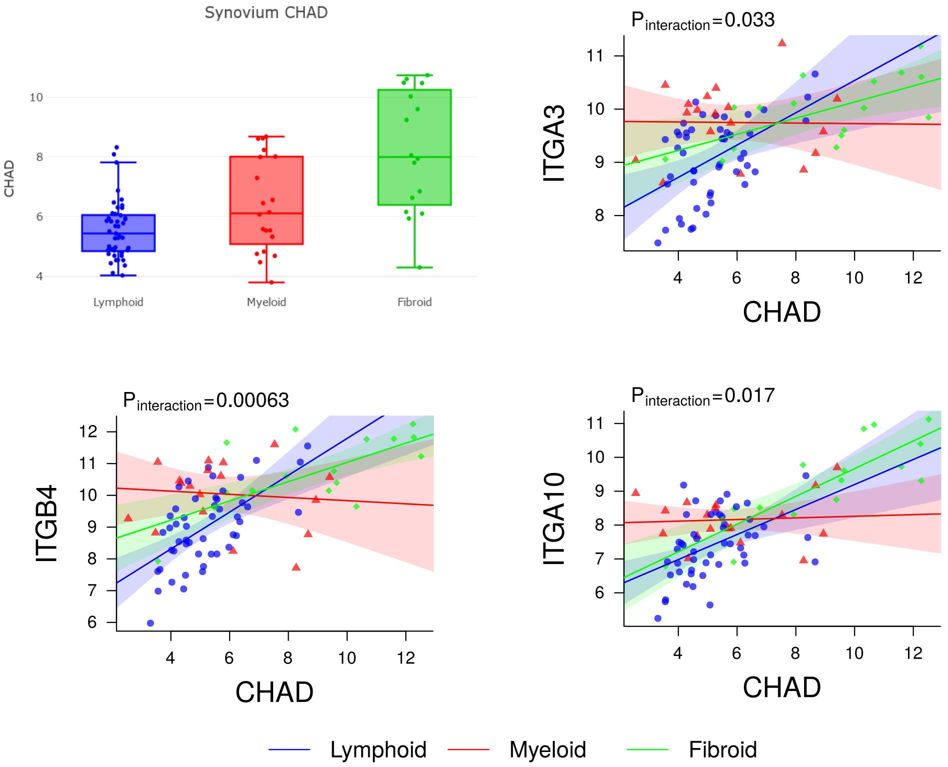


**Figure S5.** Chondroadherin (*CHAD*) shows different levels of expression across pathotypes and its interaction with multiple integrins reflects differential correlations between the histological subgroups. Statistical analysis performed via robust linear regression with interaction term. P values of the gene:pathotype interacting term are shown on top of each regression plot.

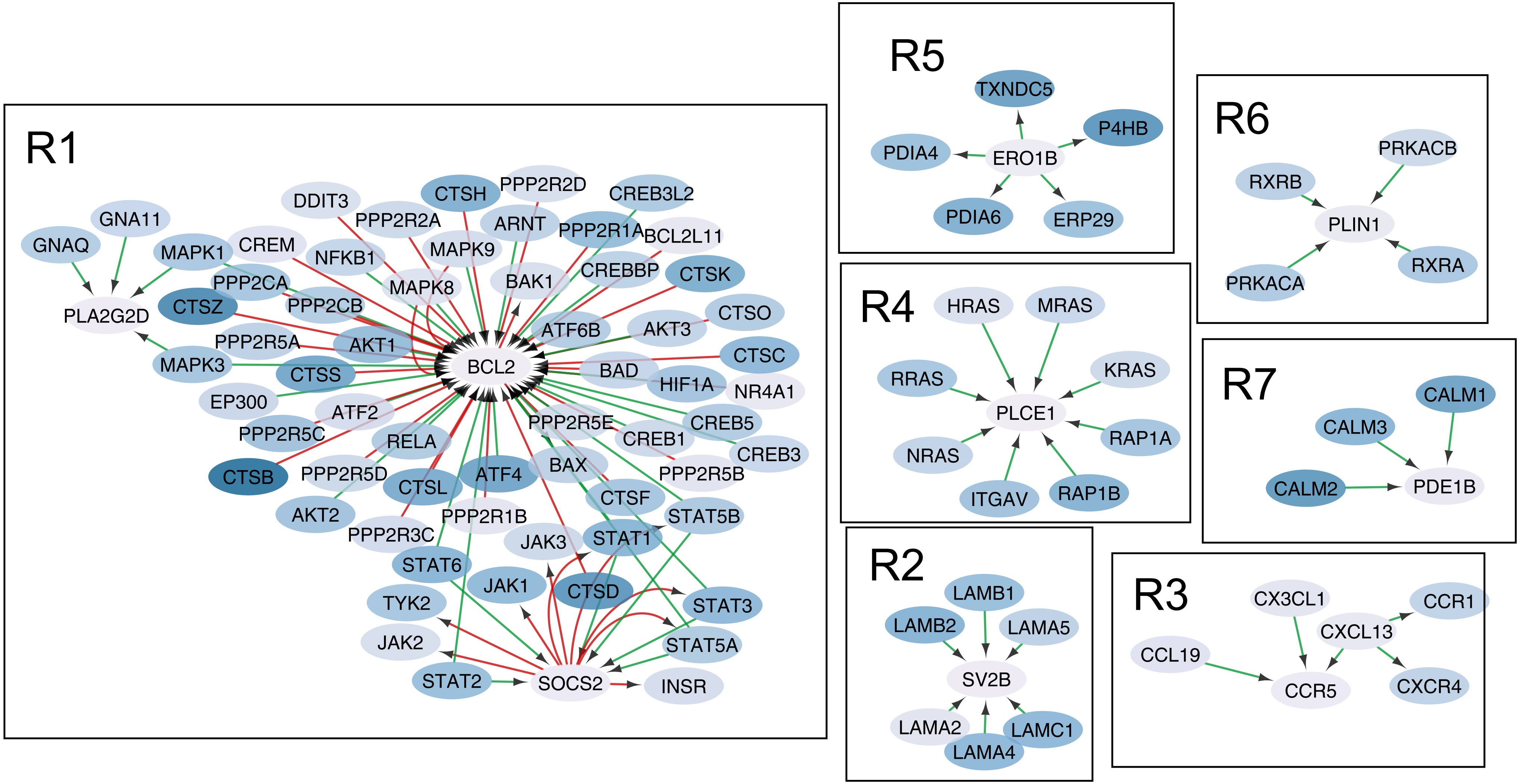


**Figure S6.** Network of specific, active interactions of the good responders group compared to the poor responders. The network contains 107 nodes and 109 edges, grouped in 7 clusters. Interactions classified in KEGG as activation links are showed in green. Inhibition links are showed in red.
The most prominent clusters of this network are reported in more detail in Figure 3A.

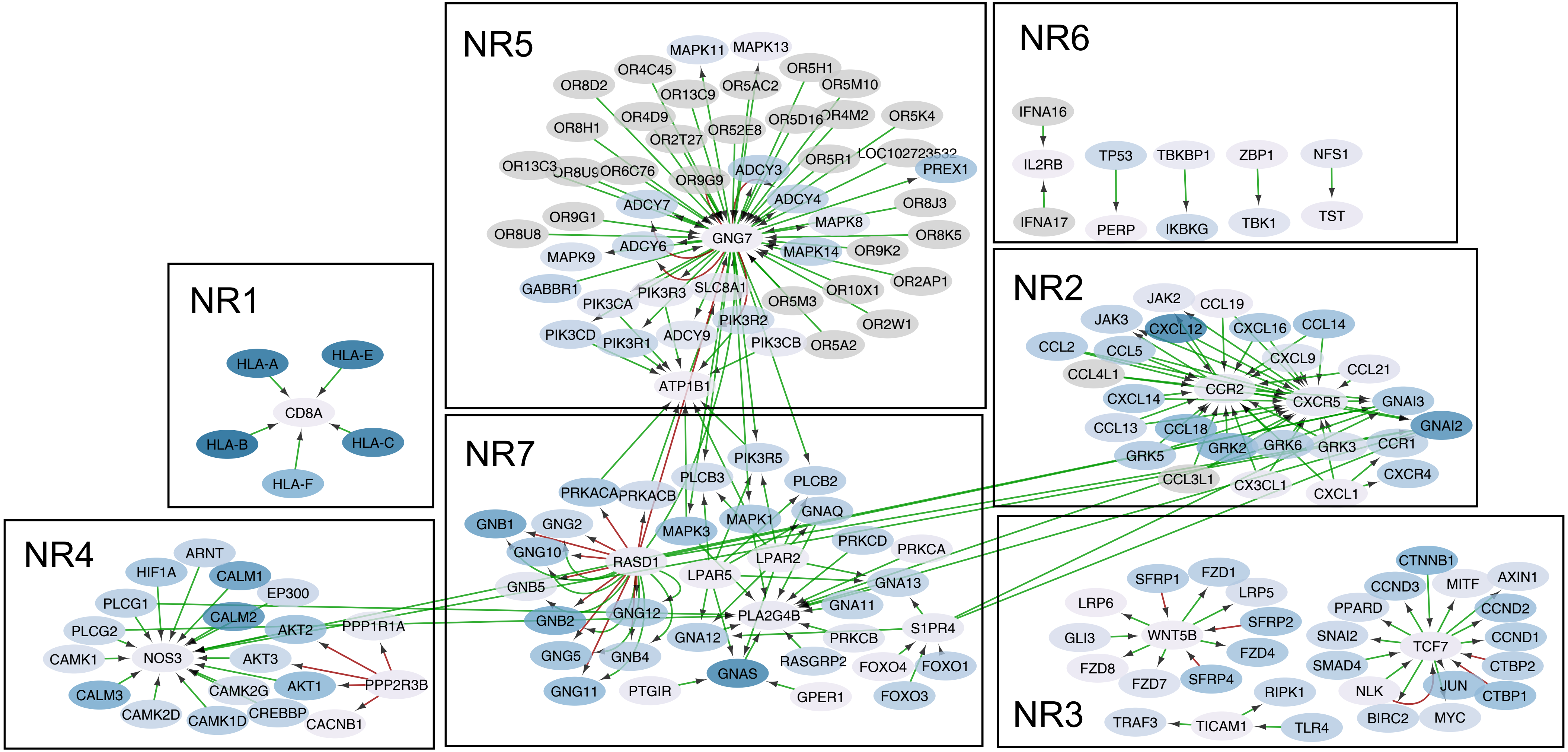


**Figure S7.** Network of specific, active interactions of the poor responders group compared to the good responders. The network contains 180 nodes and 242 edges, grouped in 7 clusters. Interactions classified in KEGG as activation links are showed in green. Inhibition links are showed in red.
The most prominent clusters of this network are reported in more detail in Figure 3A.

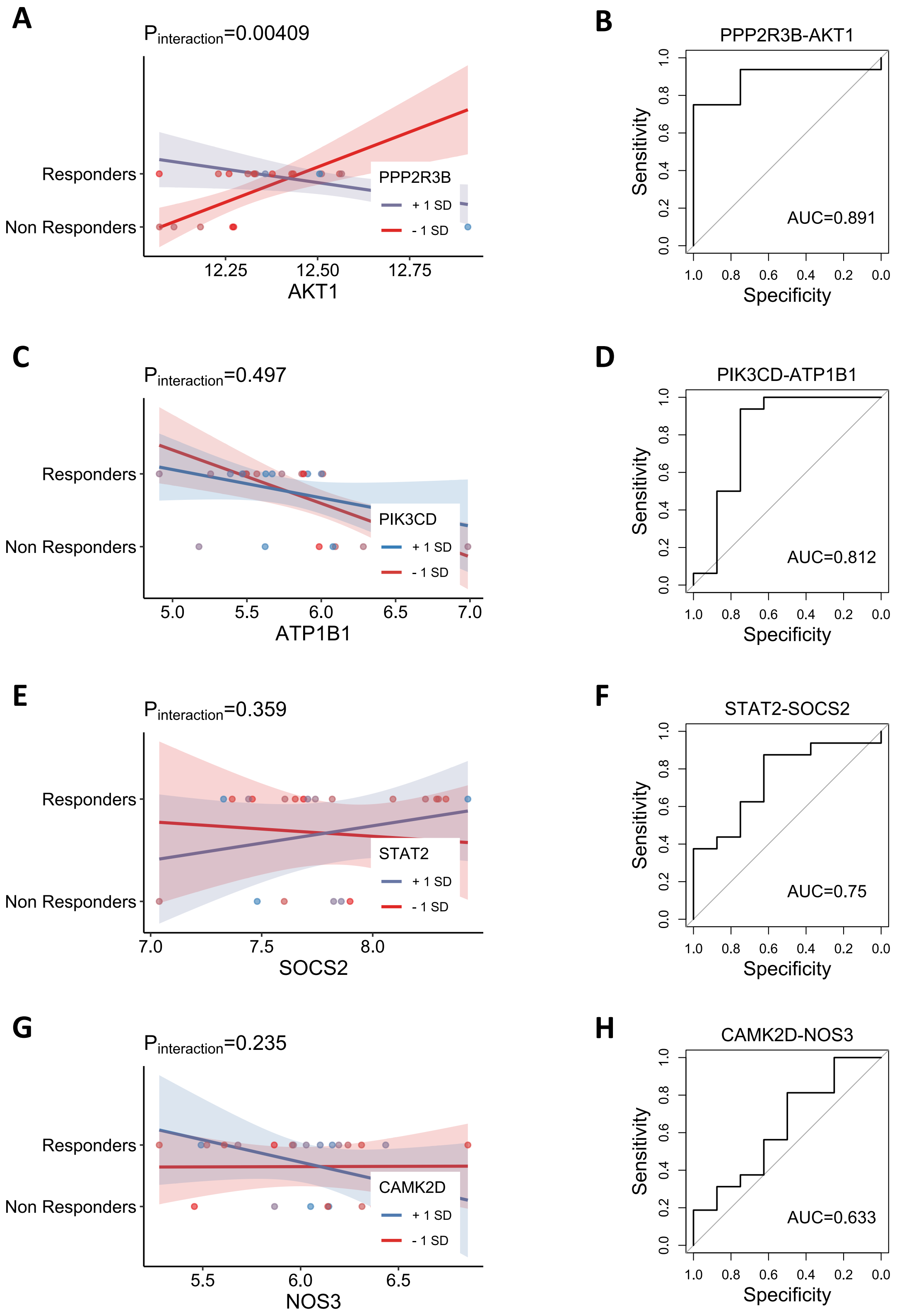


**Figure S8**. Validation of predictive models derived from synovium and tested in whole blood samples. **(A, C, E, G)** Logistic regression of response as a function of **(A)** *PPP2R3B* and *AKT1* **(C)** *PIK3CD* and *ATP1B1* **(E)** *STAT2* and *SOCS2* **(G)** *CAMK2D* and *NOS3.* P-values of the response:gene interacting term are shown. Expression of the second gene is dichotomized at ±1 standard deviation. **(B, D, F, H)** Receiver operating characteristic (ROC) curve analysis of the of robust linear model ability to predict response using **(B)** *PPP2R3B* and *AKT1* **(D)** *PIK3CD* and *ATP1B1* **(F)** *STAT2* and *SOCS2* **(H)** *CAMK2D* and *NOS3*.
